# Author response: It currently remains uncertain whether early benefits of transplant *versus* dialysis differ in ethnic minorities

**DOI:** 10.1093/bjs/znae114

**Published:** 2024-05-08

**Authors:** Daoud Chaudhry, Felicity Evison, Adnan Sharif

**Affiliations:** Department of Surgery, University Hospitals of North Midlands, Birmingham, UK; Data Science Team, Research Development and Innovation, University Hospitals Birmingham, Birmingham, UK; Department of Nephrology and Transplantation, University Hospitals Birmingham, Birmingham, UK; Institute of Immunology and Immunotherapy, University of Birmingham, Birmingham, UK

We thank Tingle and colleagues for their interest in our article^1^. They raise an interesting point concerning our manuscript, which they interpreted as suggesting that the benefit of kidney transplantation is delayed in ethnic minorities and may be incongruent with the actual data.

For clarity, our conclusion states: ‘proceeding with transplant surgery afforded a survival advantage regardless of age and/or ethnicity, but the benefit was deferred for some demographics (for example older patients from ethnic minorities)’^1^. In our opinion, we felt this observation was likely due to low numbers, as highlighted in the manuscript, rather than a genuine biological effect. Tingle and colleagues suggest that this observation may relate to limitations of the specific analytical technique and suggest an alternative method. However, we believe their suggested method would only look at modifying results by ethnicity for the treatment variable (that is transplantation). To perform this analytical technique robustly, we need to understand the impact across all variables and there are insufficient data to do that. In addition, although there is significant debate about whether adjustment for multiple comparisons should be performed, most statisticians agree that the Bonferroni correction as performed in their *Table 1* should no longer be used. Limitations exist to all statistical approaches, but our methodology is the most used.

Fundamentally, the intention with our original article was to challenge any perception bias that ethnic minorities and/or older kidney transplant candidates do not attain the same survival benefit after kidney transplantation *versus* remaining on dialysis. Our data categorically refute this and encourage a broadening of eligibility for kidney transplant assessment. On this issue, we are both in agreement. Ethnic minorities already face inequity of kidney transplantation access and the correct interpretation of our data should ensure that all eligible patients with kidney failure receive the same opportunities for kidney transplant surgery as anyone else.
